# UK local government experience of COVID-19 Lockdown: Local responses to global challenges

**DOI:** 10.1177/02690942231181562

**Published:** 2023-06-08

**Authors:** Richard Machin

**Affiliations:** 6122 Nottingham Trent University, UK

**Keywords:** COVID-19, local government, emergency planning, local governance, partnership work

## Abstract

Local authorities in the United Kingdom played an integral role in responding to the COVID-19 pandemic. During a time of unprecedented crisis, local authorities acted quickly to strengthen local governance arrangements and to deliver frontline services.

The public health emergency created unparalleled challenges for local authorities. This paper reflects on the experience of Nottingham City Council, a local authority in the East Midlands of England. It draws on an in-depth interview with the then Deputy Leader of the authority and the chair of the Outbreak Control Engagement Board, and the publicly available minutes from this committee. This paper places the specific experiences of this local authority in a broader context reflecting on emergency planning, best practice, and legacy. It contributes to understanding the national impact of COVID-19 on local government.

Four key themes emerge from this analysis: first central government was too slow in responding to the emerging crisis and communication with local government was poor, second the local authority quickly moved online but there has subsequently been a return to pre-pandemic working practices, third local government played a critical role in supporting vulnerable citizens despite huge budgetary pressures, and finally relationships between partners was crucial in achieving positive outcomes.

## Introduction

COVID-19 impact has had a profound impact on local government in the UK. The aim of this paper is to convey the experience of one local English local authority, Nottingham City Council, primarily during the first national lockdown of March to June 2020. Thematic analysis was used to evaluate an in-depth interview with the then Deputy Leader of the authority and chair of the Outbreak Control Engagement Board, and the publicly available minutes of this committee. Four key themes emerged from this analysis which are set out in the above abstract.

Nottingham City Council is one of England’s 62 unitary authorities with responsibility for providing all local government functions in the city. This arrangement differs to the areas of England with two tiers of local government; in these localities, service delivery is split between a county council and a district council. Nottingham City Council is represented by 55 elected councillors in 20 wards. The last local election in May 2019 resulted in the Labour group retaining control of the Council with 50 councillors; the Conservative Group have two Councillors and there are three independent elected members (Nottingham City Council, 2022). The 2021 census estimated that Nottingham has a population of 323,700.

The remainder of the paper takes each of the four identified themes and reflects on the experience of responding to the pandemic on a local level; key quotes from the Deputy Leader underpin this analysis. The unique experiences of this authority are placed in a wider context; each section considers issues of broader national significance, and longer term legacy.

• Central government was too slow in responding to the emerging crisis and communication was poor

On 25 March 2020, the Coronavirus Act received Royal Assent, two days earlier the then UK Prime Minister, Boris Johnson, had announced the first UK lockdown, instructing the UK population to ‘stay at home’. The timing, extent and impact of the initial UK lockdown are intensely contested; the view of the Deputy Leader of Nottingham City Council at this time is clear:

‘We should have gone into lockdown quicker. There was a battle going on between personal freedom and the collective good’

There is an acceptance of the unprecedented and formidable nature of the challenges facing central government, but a clear criticism of the ideological stance which underpinned decision making. There is a belief that an emphasis on personal freedoms, the embracing of a libertarian approach, conflicted with the responsibilities of a local authority to serve and protect citizens. The elected member interviewed stated that there was concern from public health professionals, both locally and nationally, about the approach adopted by central government. There is a clear view that this political stance, an unwillingness to move more decisively and quickly, led to hardship and ultimately unnecessary deaths:

‘I'm absolutely sure that we would have saved more lives’

There is a compelling body of evidence that supports the above view, this centres on the belief that the ‘go hard, go fast’ approach to COVID-19 lockdowns was effective; New Zealand and Iceland are cited as success stories (Lewis, 2022). In the UK, the prominent epidemiologist, Neil Ferguson, notably stated that deaths would have been halved if the UK had entered lockdown a week earlier (Stewart and Sample, 2020). When the World Health Organisation officially declared COVID-19 to be a pandemic it emphasised that countries had significant influence in stopping the spread of the disease but that a ‘whole government-whole society’ approach was needed (Mahase, 2020). Studies show that lockdowns were key to limiting infection rates and protecting intensive care units from being overwhelmed (e.g. see Davies et al., 2020). However, assessing the value and timing of a lockdowns is a not a binary exercise, there are many complicating and country specific factors such as pre-pandemic levels of government financial support, compliance with social distance rules, population demographics and housing status. On these factors, a clear view was expressed that a decade long programme of austerity had left Nottingham in a compromised position to respond to the pandemic:

‘Bearing in mind we’d have many years of budget cuts as it was, and we were in financial difficulties. It’s a problem, a national issue. It's particularly affected big cities.’

Lewis (Lewis, 2022, p4) states that ‘Lockdowns hold another clear lesson: they exacerbate inequalities that already exist in society. Those already living in poverty and insecurity are hit hardest.’ Long-term austerity combined with a pandemic is a particularly toxic combination for local authorities such as the one which is the subject of this commentary which ranks as 11 out of 317 authorities in England on the Indices of Multiple Deprivation (housing, health and well-being, education and skills, income deprivation, crime).

During the initial lockdown period in spring 2020, daily leadership meetings were held by Nottingham City Council to coordinate decision making and the response to the pandemic. It is held that these meetings were made more challenging because of the inconsistent, and often partial data and communications being shared by central government:

‘Some days it was completely unclear what was going on. The latest information came out in a pretty bitty way. So, there’ll be an announcement that there was going to be support for businesses. But you didn’t know how it was going to be delivered. You didn’t know when the money was coming. You didn't know how you were going to get it out and the systems had to be developed really quickly to respond.’

There is evidence that this local authority’s view of inferior central government communication was shared by a significant number of the general population. Ipsos MORI polling in May 2020 showed a reducing satisfaction with the government’s communication strategy as the first lockdown persisted; 43% of respondents stated that coronavirus messaging was not very/at all clear (Ipsos MORI, 2022). Similarly, an online focus group study conducted in March/April 2020 reported a lack of trust in the government and lack of clarity around social distancing and stay at home requirements (Williams et al., 2020). Thematic analysis of the prime minister’s public communication also exposed a complacency and lack of preparedness in dealing with the first 6 months of the pandemic, and unfavourable comparison were made with the communication strategies of high reliability organisations, such as nuclear power stations or aviation companies (Sanders, 2020).

A key example of the tensions between central government messaging and the efforts of the local authority to deliver strong public health directives came later in the pandemic (August 2020) with the Eat Out to Help Out policy:

‘I just found it really frustrating. We were desperately trying to get the public health measures across and to hold the line and make sure that we were doing the right thing and trying to protect our citizens as much as we could. And it felt like they were just undermining you all the time’.

Over 160 million meals were claimed through this policy (González-Pampillón et al., 2021), with increased hospitality footfall (5–6%) and recruitment in the service sector (7–14%). However, the public health concerns were well-founded as there was an identifiable increase in COVID-19 cases in areas with a high participation rate in the scheme (Fetzer, 2022).

The central to local government communication dynamic became particularly problematic when lockdown restrictions were eased. Tensions were apparent as Westminster were eager to emphasise a return to normality, but the local approach was notably more cautious:

‘The government's approach is this is finished to the extent that you are not actually getting data; so that shows that they want to think this is a historic thing it's happened and just move on from it. We were absolutely clear that we wanted to continue with the messaging about mask wearing and distancing to just to keep it on the radar, be cautious, be safe’.

• Local authority services quickly and efficiently moved online, there has been a gradual return to pre-pandemic working practices

‘The emergency planners were taking it seriously and we were being sent home before the government had said go home. We go home today, and we don’t know when we will be coming back’

In common with other areas of the public sector, the experience described by this local authority was that the COVID-19 pandemic greatly accelerated digital and home working practices. The post-2010 local government budget cuts had necessitated ‘new ways of working’ which often involved some home working and hot desking, but not the wholesale home working arrangements that the pandemic demanded. The view held by the elected member interviewed for this commentary was that homeworking was managed in an efficient manner and that services quickly adapted to digital platforms:

‘It was all hands to the pump; it went much more smoothly than anybody could have expected’

A distinction can be drawn between the challenges faced during the first lockdown for senior decision makers and managers, such as elected councillors, and front-line staff. For more senior staff lockdown necessitated the rapid introduction of digital networking, online meetings and ensuring systems were put into place to ensure that staff were equipped with the appropriate technology and online line management support to deliver services to the public.

For the elected members at the local authority which is the subject of this commentary, key online decision making and information sharing forums were the COVID-19 Outbreak Cell (which met daily) and the Outbreak Control Engagement Board (meeting 2–3 times per month). The Outbreak Control Engagement Board represented by a combination of local authority, NHS, and Public Health professionals was responsible for Nottingham’s overall COVID-19 outbreak control plan. Members of the Outbreak Cell included senior local politicians, local Public Health, and NHS Trust Infection prevention and control (IPC) leads, Environmental Health, and local authority communications teams.

Drawing on national data (for example, pillar 1 and 2 COVID-19 statistics) and local data (e.g., care home information) the Outbreak Cell was responsible for co-ordinating incident management teams which responded to issues arising in hotspot or high-risk areas. Key functions of the Outbreak Cell were to compile a live situation list identifying required actions to respond to emerging pandemic issues, to produce deep-dive reports using all available data, and to forward plan with data analysis of lessons learnt to feed into an outbreak control plan.

These online committees were rated as being highly effective, delivering high-level strategic and operational outcomes through online networking:

‘There was a lot of cooperation between officers and councillors. There was a lot of problem solving and forward planning that went on that was really good.’

The overall local outbreak control plan governance structure, including the relationship between the Outbreak Control Cell and Outbreak Control Engagement Board, is shown below:


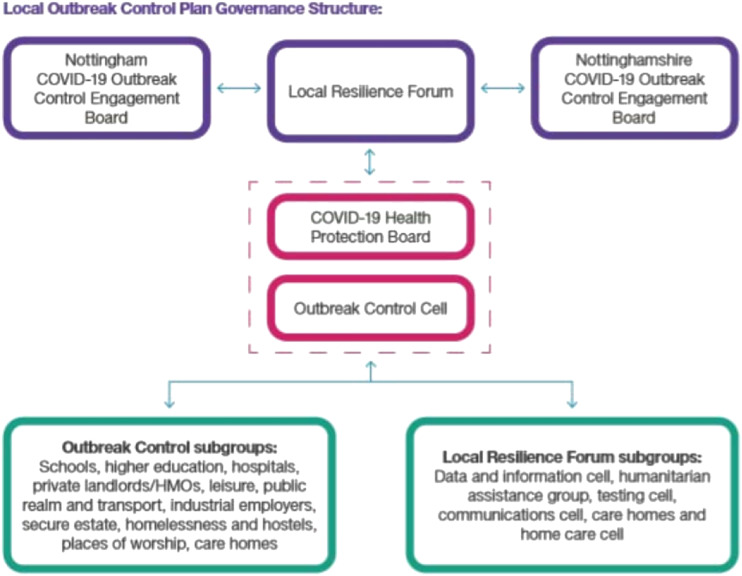


The challenges facing front-line local authority staff, responsible for delivering services directly to the public, were somewhat different in nature. The adjustment to home working and relying on digital communication posed similar issues as for other staff, but the responsibility to protect and engage with citizens presented unique issues. An important element of face-to-face working with the public (through home visits or office appointments) is the assessment of need, weighing up visual cues, assessing environmental factors, developing ongoing relationships where important information or vulnerability can be shared.

Clearly, during COVID-19 lockdowns this sustained and personalised way of working became challenging. Initially, this was compounded by complex practical issues where systems needed to be quickly developed which could ensure digital safety and data security (e.g., around the verification of correspondence, eviction notices, and social security claims). In the absence of face-to-face appointments, there was a reliance on telephone communications, which were appropriate for some citizens but not all. Front line staff needed to utilise text and video relay services for deaf service users, and language interpreting services for those whose first language is not English. Effective community engagement assumed an even more critical role than in the pre-pandemic period, as did effective communication strategies.

For Nottingham City Council, reported safeguarding concerns fell by 24% for the period April 2020–March 2021; safeguarding and quality proceeding for care homes fell by nearly a half in the same period. During lockdown abuse and neglect was concealed as a result of social isolation, and the cessation of usual community services and monitoring (Nottingham City Safeguarding Adults Board, 2021). Home visits were only conducted where cases were classified as high risk; statutory support usually delivered in care homes was significantly reduced. During this period, this local authority moved to provide adapted ways of working: electronic newsletters to practitioners, virtual training sessions on Microsoft Teams, online delivery of the Complex Persons Panel, citizens experiencing domestic abuse were commonly communicated with through mobile technologies, virtual quality monitoring tools to assess care home provision and a COVID-19 Taskforce comprising the local authority, Care Quality Commission and Clinical Care Group.

‘It feels like a permanent hybrid working environment for most people apart from frontline staff. There is a wish to speed things up decision making but in that respect, I don't think things have changed fundamentally.’

In the post-pandemic period, particularly following the high uptake of the COVID-19 vaccine, hybrid working, and the utilisation of digital technologies have endured for this local authority, although in-person contact has not returned in full for all services. Evidence from this case study shows that there has been limited changes to internal decision-making processes, or the speed at which decisions are made. Typically, there is a desire for CPD and localised training to return to a face-to-face delivery format. COVID-19 practices have endured where they have worked effectively and demonstrated mutual benefits for the local authority and citizens.

Ultimately local authorities need to decide which ways of working will continue in the longer term and adopt a nuanced and blended approach to service delivery, mindful of the fact that digital exclusion is an element of contemporary social deprivation experienced by many citizens (Holmes and Burgess, 2022). To navigate this period the principles of trust (between citizens, staff, and managers), reciprocity (considering the implications of how home environments become a shared space of work), and ethics (considering what is the appropriate action to take), as established by Kingstone et al. (2022) should be paramount.

The experience of front-line staff at Nottingham City Council largely accords with the wider research on homeworking during the pandemic. This has found that home workers quickly developed digital skills and appreciated the absence of work commuting (Al-Habaibeh et al., 2021). Overall productivity increased although this was less prominent for workers who were home-schooling; the majority of workers missed the face-to-face contact with colleagues and informal office conversations (Deole et al., 2023).

• Local government played a critical role in supporting vulnerable citizens despite huge budgetary and logistical pressures

The COVID-19 pandemic served to highlight the critical role local government plays in supporting local communities and residents. This is even more remarkable given the compromised position that local authorities found themselves in when the pandemic hit, due to the sustained programme of austerity. Gray and Barford (2018) emphasise that central government funding cuts have not only reduced the capacity of local government, but created geographic inequalities, a feature of which is ‘urban austerity’. Cities such as Nottingham, with high levels of poverty and a declining traditional industry base, have a high reliance on central government grants and are particularly susceptible to reductions in funding. Ahrens and Ferry (2020) contend that central government pandemic emergency planning focussed on health to the exclusion of local government, but that local government responses have been critical in the COVID-19 recovery period. Between March and October 2020, English local authorities received £4.61 billion emergency funding based on the severity of local lockdown restrictions, per capita and adult social care spending, and a relative needs formula accounting for deprivation and the cost of delivering services. Gore et al. (2021) argue that although this central government funding was significant, an underlying ambivalence towards local authorities in England (not present in the devolved nations) was displayed by locking them out of testing and contact tracing services.

In common with other local authorities, Nottingham City Council had to quickly develop and deliver a wide range of services to meet COVID-19 community needs:

‘In order to provide that support, the sensitive support to deal with people who were in a particularly vulnerable place during lockdown, medication or food or social support. All those systems had to be created from scratch. We were dealing with all sorts of other sorts of complicated social and health issues’.

As well as maintaining statutory services, a new range of duties were delivered including ensuring the supply of personal protective equipment (PPE) reached frontline services, supporting local businesses, delivering a customer hub ‘golden number’ to support people who were self-isolating but did not have social networks to collect medicine or food, supporting those who were street homeless into accommodation, and working with schools to deliver online learning and in-person teaching for vulnerable children and children of critical workers.

To be able to deliver these new and complex range of services required local authority staff to demonstrate a high level of flexibility while at the same time experiencing enormous internal pressures:

‘We were worried because there was such a high level of absence. At the same time, we needed more people to be employed in care jobs. We were trying to redeploy people into key roles, people from libraries were being asked to go into caring roles, for example’.

Meeting the needs of the community required an adaptability, both in operational and personnel terms:

‘When we set up the ‘golden number’, there was a lot of training for those operators who were suddenly working from home rather than in Loxley House (Nottingham City Council’s customer service hub)’

Balancing the needs of the citizens and the community, the safety of local authority staff, while maintaining statutory services was challenging. An example of this was the challenges around waste disposal:

‘To keep people healthy, it is crucial not to have loads of waste on the streets and they normally have a driver and two people in the cab but because of social distancing rules, we couldn't have three people in the cab, only one with a vehicle following up. The amount of commercial waste that would normally be coming out of businesses and offices and workplaces dropped right down, but the domestic waste went up hugely. So, it was a juggling exercise. People that would normally be cleaning the streets and working in parks etcetera were brought in to try and keep the bins going’.

In the early stages of the lockdown, one of the most pressing challenges for local authorities was procuring and supplying the appropriate level and quality of Personal Protective Equipment (PPE). This was much publicised at the time and has subsequently been the subject of significant scrutiny. Between February and July 2020, the Department of Health and Social Care (DHSC) spent £12billion on 32 billion items of PPE and maintain that supply lines were never exhausted. However, concerns were voiced from within the NHS that staff were not provided with PPE which met Public Health England standards, and that inadequate supplies put staff at unacceptable and unnecessary risk of infection (Oliver, 2021). The National Audit Office (2020) found that the government’s emergency procurement processes failed to meet acceptable standards and public confidence was compromised over perceived conflicts of interest and retrospective awarding of contracts. Similarly, the Public Accounts Committee (2022) found that £1.3 billion was spent on PPE without treasury approval, that 24% of contracts awarded were subject to legal challenge, and that equipment was purchased which failed to meet requirements and was poor value for money.

These logistical problems were acutely experienced on a local level:

‘Towards the end of March 2020, we were waiting for supplies. And there was talk of some PPE coming from China but there were customs issues. There were 225 thousand masks waiting in China that we'd ordered and none of it was getting here. This was discussed daily, and we were trying to find local suppliers. We were giving out information to care staff about how to work without PPE safely. The NHS didn’t have enough, so they couldn't share it. It did begin to ease off at the start of April 2020 when we got national supplies’.

The scale of the COVID-19 support operation in Nottingham City was impressive. This included distributing over 1.9 million items of PPE, knocking on the doors of nearly 2500 residents who were not responding to phone calls, telephoning 12,000 people were shielding and providing 2000 emergency food parcels. However, the financial impact of the pandemic was enormous, costing this local authority around £90 million in increased spending and lost income from sources such as leisure centres and car parks. Central government funding amounted to £23.5 million meeting only 27% of the new COVID-19 burdens. This funding shortfall necessitated funding cuts of £12.5 million during the financial year 2020/21 resulting in the deletion of 154 full-time posts, the closure of a day centre for people with disabilities, delaying an apprenticeship programme and closing several play areas.

• Relationships with and between partners was crucial in achieving positive outcomes

‘There was a lot of a lot of cooperative work which went on. I think people rallied around tremendously. Partner organizations, council staff, volunteers. Lots of people went well beyond the expectations of their role’.

Local authorities can only effectively meet the needs of the communities they serve if their functions are underpinned with effective partnership work. Partnership work with agencies from the public, private and voluntary sectors was mainstreamed during the New Labour administration (Wilkinson and Craig, 2002). Effective partnership work has long been recognised as an integral component of combating poverty and social exclusion, encouraging regeneration, and promoting active citizenship (Pearson, 2001). Despite these laudable aims, Balloch and Taylor (2001) identify challenges to partnership of a political, cultural, and technical nature. During the pandemic, two-tier authorities arguably experienced greater challenges in partnership working than unitary authorities, needing to manage an additional level of collaboration.

For Nottingham City Council, an effective response to the pandemic relied on partnership work with a wide range of internal stakeholders (e.g., public health and housing) and external stakeholders (e.g., NHS, the media, and local universities). The experience of the Deputy Leader of the Council was that positive outcomes were identified when working with all partners, but there were some identifiable differences in the dynamics of the partnership arrangements. Overall, partnership working with internal partners, where there were long-term and mutually beneficial outcomes, was seen as more straightforward than partnership with external partners where a higher level of negotiation and compromise was sometimes required.

‘Around the neighbourhoods, which are particularly diverse, additional signage was paid for through the public health grant, about mask wearing and social distancing in many languages. We also started a regular e-mail (Monday, Wednesday, and Friday) as part of our attempt to reach more people about keeping safe. This was very successful in reaching a lot of people and had a very high level of engagement’.

The above quote demonstrates the positive relationship between elected members and Public Health Nottingham City. This relationship was critical in the local delivery of public health messaging, particularly given the experience of problematic and inconsistent central government communications described earlier. Local politicians were able to utilise local public health intelligence and harness their own knowledge of local communities to deliver tailored and culturally appropriate communications. Health campaigning during the COVID-19 lockdowns was able to draw on local authority-public health relationships established through the statutory Health and Wellbeing Board and shared Strategic Health Framework.

‘I used those meetings very much as a way of getting the public health message out to the media and the public and for different organisations to showcase what they were doing. For example, environmental health did a lot of work with businesses, shops, and takeaways about how to be COVID-safe, making sure they were displaying the correct information’.

The importance of the Nottingham COVID-19 Outbreak Control Engagement Board has been described in an earlier section of this paper, and it is a key example of effective partnership working. The pandemic necessitated multi-sectoral decision making and responsibility between local politicians, and representatives from health, public health, social care, education, and housing. It is the view of the elected member interviewed for this paper that the board demonstrated best practice partnership work in all its core functions. These include data sharing, health messaging, promotion of services, engagement with the public and media, high-level analysis of national and local statistics, risk management and emergency planning, and financial decision making.

The elected member interviewed for this paper believes that the partnership work with education and housing stakeholders during the first COVID-19 lockdown was effective and helped to mitigate the impact of the pandemic on children and young people and those in housing need.

‘We often had representatives from the education service there to report on what was happening because it was a big concern and schools did a marvellous job in managing the outbreaks’.

The local education sector faced a huge range of challenges during the first lockdown. In May 2020, Nottingham Centre for Children, Young People and Families raised concerns about a lack of clarity about national policy which was then hard to operationalise on a local level, increased and emerging vulnerability for many pupils, a heightened attainment gap between children, and concerns about remote learning and digital exclusion (Paechter et al., 2020). While the pandemic has had an enduring impact on the socio-emotional wellbeing of pupils (Tracey et al., 2022) it is the view of the then Deputy Leader of Nottingham City Council that effective partnership work and utilisation of existing relationships was crucial in mitigating some of the most damaging impacts of school closures. In practical terms, this meant an extension of the traditional role of education to include home visits for welfare checks, provision of food, delivering learning materials to pupils’ homes, and close liaison with social services where there were safeguarding concerns.

Perhaps, the clearest example of effective emergency COVID-19 policy which was rolled out nationally but relied on successful delivery by local stakeholders is the ‘Everyone In’ initiative:

‘The work to get homeless people off the street made a massive difference. People were moved into hotels which were not in use at the time and additional support was provided. It demonstrates that if you’ve actually got the funding, you can make a massive difference’.

On 26 March 2020, the government launched the ‘Everyone In’ initiative which aimed to provide emergency accommodation for rough sleepers to protect them and the public from COVID-19. Local authorities sourced hotel rooms, hostels and student accommodation and provided food, medical care, and support. By January 2021, 11,263 people had been provided with emergency accommodation, and 26, 167 had moved onto settled accommodation; in Nottingham 168 people were accommodated through the scheme (Cromarty, 2021). Independent evaluation of the ‘Everyone In’ scheme in Nottingham concurs with the positive view expressed by the elected member interviewed for this paper: ‘the unthinkable can work’ (Kaur et al., 2021). Existing relationships in the public, private and voluntary sector were utilised to provide personalised care planning including a recovery network, GP registration, homeless health team, and health shop. The real risk is that ‘Everyone In’ will be remembered only as a ‘policy pause’ it was clearly shown that adequately funded local partnerships can tackle deep-seated social problems, and there are calls for the permanent establishment of a multi-disciplinary team, commissioned by Nottingham City Council to support rough sleepers (Kaur et al., 2021).

It should be recognised that professional relationships during times of crisis are not always straightforward. Elected members must balance the demands of representing their local area and developing council policy. Pandemic decision making inevitably involved respecting a wide diversity of views. An important point of debate between the local authority and the NHS related to the location of vaccination centres:

‘We kept having discussions with them (the NHS) about how they were going to get into our diverse communities and roll out the vaccination program. It was quite a challenge at times to get them to take the program to the people if you like.’

The local authority view was that the proposed vaccination centres were geographically inaccessible and in areas which risked low engagement with key minority ethnic groups. The Council was able to help them to develop a plan so that a wider range of locations were chosen to host vaccination centres, including more temporary sites at cultural centres and places of worship. Delicate relationship management was required here to respect the views of health professionals while at the same time meeting the needs of citizens.

## Conclusion

This commentary has demonstrated the profound impact of the COVID-19 pandemic on one local authority in England and has highlighted the impact of the complex relationship between central and local government, the ability of local government services to quickly move online, and the importance (and complications) of utilising existing professional relationships and developing new partnerships.

The dominant theme emerging from the evidence gathered for the paper is that local authorities play a vital role in meeting the needs of the community; this was underscored by the swift mobilisation of services at a time of unprecedented emergency and against the backdrop of austerity. Beland et al. (2022) emphasise that a crisis such as a pandemic simultaneously aggravate existing inequalities and create new vulnerabilities; the intervention of local authorities is critical in these situations. Some of the experiences discussed here are location specific, reflecting the regional variation in need and provision, but the presiding influence of central government policy and financing is a universal experience shared in all localities. Lying beneath this control from Westminster is a disparate network of local authority provision; a debate about the appropriacy of English devolution is outside the scope of this commentary but remains a live issue.

The longer-term impacts of the pandemic on English local authorities will emerge in the coming years. Hogan et al. (2022) suggest that COVID-19 has not produced fundamental changes in state activity but should instead been seen as a ‘path clearing event’ which accelerated change and trends already in place. This analysis indicates that hybrid working arrangements are likely to become a permanent feature and there are opportunities to consolidate the partnership work and local governance arrangements which proved effective during the pandemic. The financial uncertainties faced by local authorities in the last decade will endure. The Community Infrastructure Levy (CIL) model introduced in 2010 proved to be untenable during the pandemic. This assumed that councils would raise revenue from developers completing building projects in their locality and allow investment in infrastructure or commercial assets. The Autumn Statement of November 2022 (HM Treasury, 2022) protected local government spending for the next two financial years, but severe cuts in 2025/26 will leave a funding gap of £7–9 billion. English local authorities have been given increased powers to raise council tax by up to 5% but this local discretion creates inequalities (the most prosperous authorities can generate the highest revenues) and dilemmas (are local authorities prepared to increase tax burdens during a cost-of-living emergency). Local authorities have shown great resilience during the COVID-19, but the post-pandemic landscape remains precarious.
